# Clinic Atlanta Medical College

**Published:** 1876-03

**Authors:** A. W. Calhoun

**Affiliations:** Professor of Diseases of the Eye and Ear


### Clinic Atlanta Medical College.

A. W. CALHOUN, M.D., Professor of Diseases of the Eye and Ear.

(*Continued from page* 673 *of February Journal.*)

CASE XXXIV—*Presbyopia* (*far-sightedness*) *corrected by glasses.* J. W., colored, cook-woman, Atlanta, æt. forty-eight, complains that she is unable to do fine work, or to distinctly see objects that are close to her, such as reading and sewing, or attending to some of the minuter duties connected with her avocation; and when she forces herself to continue the effort, at reading, for instance, she finds that the eyes begin to weep and burn slightly, and in a short while the letters or objects become blurred or dimmed, and she is forced to give up the work for the time, by reason of the extremely unpleasant, if not painful, sensation that ensues in the eye-ball itself. After resting the eyes for a variable time, she is again able to resume such work, to be in turn compelled to suspend the use of the eyes for the same reasons before mentioned. In threading a needle, she says, she notices that every year it must be pushed farther and farther from her, in order for her to see its eye; and now the needle is placed so far off that it is impossible for her to thread it.

Ophthalmoscopically and externally examined, there is no trace of disease, the structures being in a very healthy condition. The trouble is known as presbyopia, and is the result of age. Upon an average, at about the forty-fifth year the cornea begins to flatten and the lens to harden (retaining its transparency), as well as also to become a little flatter; the ciliary muscle begins to weaken, and the humours of the eye, to some degree, lose their refractive power. This gradually increases year by year, until the individual is forced to seek assistance. The result of these changes is that the ciliary muscle is unable to render the lens sufficiently convex to bring the rays of light to a focus upon the retina, without which the vision cannot be perfect.

The treatment, then, consists simply in assisting nature, and this is done by adjusting convex glasses, of suitable strength, to the eyes, thus enabling them to focus the rays where they normally belong, viz, upon the retina, It is generally considered that presbyopia begins when the *near point* (the nearest point at which fine print can be conveniently read) is removed farther than eight inches from the eye. According to this we have an easy way of estimating the number of the desired glass. For instance, the person can read print only at sixteen inches; then 1-8—1-16=1-16, the number of the glass which enables her to read at the normal distance, eight inches. By this formula we can always estimate the proper glass, provided there is no complication. It is best, however, to commence always with a still weaker glass than the calculation shows, and then gradually increase as the necessity demands.

Glasses should be worn as soon as the presbyopia manifests itself. It is a mistake to put off wearing them as long as possible, which advice is always given by the general practitioner, not conversant with the troubles that may follow the non-use of them.

The patient was supplied with a weak convex glass (No. 40), and told to report again if she had trouble. Nothing more has been heard from her.

It is extremely seldom that negroes call for the use of glasses, simply from the fact, I suppose, that they have comparatively little necessity for the exercise of their eyes upon very minute objects.

Case XXXV—*Acute Catarrh of the Middle Ear. Cure.*—V. A., æt. seventeen, Atlanta, took cold several days ago, and to-day comes for relief from a gradually increasing deafness. There is a constant roaring or buzzing in the ears, and a feeling of fulness, with a very unpleasant sensation in the interior, now and then amounting to severe pain, extending even to all the surrounding parts. The hearing has gradually diminished, until he can now only hear very loud conversation. Upon examination with the mirror, the drum membrane is found to be slightly bulging into the external canal, and has to a great degree lost its brilliancy. There is evidently some secretion in the cavity of the tympanum, produced by the inflammation of the mucous membrane lining it. The patient has also pharyngitis, and the inflammation has traveled from the pharynx along the eustachian tubes into the middle ear, and thus set up the present trouble.

Strong gargles of alum and chlorate potash were prescribed, to be used alternately and frequently during the day, in order to relieve the pharyngitis, and his bowels kept in rather a loose condition for several days. Hot mustard foot baths were also directed to be used. Locally, warm water with morphine was poured into the ear every hour or two, keeping it up for ten or fifteen minutes at a time. The eustachian catheter was used once daily and air thrown into the tympanic cavity, and when the pain became intense, morphine was used hypodermically. Under this treatment the patient recovered in about one week, without suppuration of the ear, and a complete restoration of hearing.

Case XXXVI—*Double Strabismus—Operation.*—E. Y., æt. seven, had inflammation of the eyes when quite young, which resulted in small corneal opacites, covering, to some degree, the pupils. There is in the left eye a central capsular cataract, and a peripheral cataract in the right. Nystagmus (a dancing movement) is present in both eyes, which, in my experience, is invariably an evidence of a defect of vision, originating in some trouble in the interior of the eye.

The strabismus began when the child was about two years old, evidently produced by the slight central opacity on the lens, causing an effort to see to one side or the other, thus producing a turning inwards of the balls.

Chloroform was administered, and the internal rectus of each eye divided at its insertion into the ball, after making an opening through the conjunctiva directly over this point.

Cold salt water was applied frequently during the day, and in the course of ten days all soreness and blood-shot appearance of the conjunctiva had disappeared. As the muscles re-inserted themselves into the sclerotic, the left contracted slightly, so as to again turn the eye inward to a very slight degree. This will very probably necessitate a second operation. In a majority of such cases I find a second and sometimes a third operation necessary; but each case ought to be made a success, if both the patient and physician have courage and patience enough to carry the treatment thus far.

The vision in this case has been already very much improved, it having been materially diminished by the turning in of the balls.

Case XXXVII—*Temporary Obstruction of the Eustachian Tubes, and Middle Ear Catarrh, the result of a Cold—Cure.*—C. T., colored woman, æt. forty, Atlanta; has taken cold, producing a severe acute naso-pharyngitis, which has lasted for several days. To-day she complains of an almost intolerable roaring in the left ear with general headache, and an acute pain in the interior of the ear whenever she talks. There are also some of the constitutional symptoms usual in cases of severe cold. In the external auditory canal a small accumulatlon of cerumen exists, and the membrana tympani presents a slightly congested appearance. The mucous lining of the eustachian tubes is considerably swollen, making it difficult to enter even a small catheter into their pharyngeal openings, and air is forced along their tract with a very great deal of trouble. Her hearing is very materially diminished.

She was treated by iodine inhalations twice daily, to reduce the naso-pharyngitis, and the swelling at the mouth of the eustachian tubes. The simplest manner of taking these inhalations is, to pour twenty to thirty drops of the tincture of iodine into a cup of very hot water, and inhale the steam arising from it, heating it again and again in order to get the use of the steam. A strong gargle of chlorate potash was also used frequently during the day. By means of the syringe, the external auditory canal was cleansed, and the cavity of the tympanum and the eustachian tubes were opened by the daily use of Politzer's method of inflating the ear.

In the course of ten days she had recovered entirely, with good hearing, and was dismissed.

Case XXXVIII—*Deafness of Twelve or Fifteen Years' Duration from Inspissated Cerumen—Complete Restoration of Hearing upon the Removal of the Wax.*—Mrs. E., æt. thirty, Decatur, Ga.; says that her hearing began to fail perceptibly about fifteen years ago, increasing more and more every year until she could only hear very loud conversation close to her. The hearing power has remained in this condition for the last twelve years, varying but little while suffering from cold or recovering from it. The watch has to be placed within half an inch of the auricle to enable her to hear its ticking. She suffers no pain about the ears, the only trouble being the deafness.

Upon examining the ears with the otoscope, the external auditory canals were found to be almost filled with extremely hardened wax; so hard, indeed, that being touched with a probe, it imparted the sensation and feeling as though a very solid body, as stone for instance, had been met. The mass was so firmly impacted in the canals that it was immovable, and presented the same appearance that is usual in such troubles, viz: a very black or dark amber color.

A strong solution of bicarbonate of soda and glycerine was prescribed, and she was directed to fill the canals with it four or five times daily, after heating to a milk-warm state, and allow it to remain in the ear five to ten minutes before turning it out, after which a piece of cotton was to be placed in the canal, in order to keep in the moisture. After keeping this up for several days to soften the wax, the ears were syringed and immense quantities of it were brought away. On account of the hardness of the wax, this treatment had to be kept up for ten days or two weeks before most of it was removed, when the canals were seen to be filled with detached epidermis. This was removed with forceps, and presented a complete cast of the auditory canal and of the external surface of the membrana tympani. After the removal of these, she complained that "she heard too much"—that the sounds were painful to her—no doubt due to sensitiveness of the drum-heads. A little cotton in the ears relieved this. In testing her hearing, a day or two later, the watch was heard more than two feet from either ear, and a very low whisper could be heard across the room from her.

She was dismissed with the injunction to keep her ears washed out once a month.

Thus was severe deafness of twelve or fifteen years' standing produced by an accumulation of wax, and thus was hearing entirely restored by the softening and removal of the wax by syringing.

